# Stem Trait Spectra Underpin Multiple Functions of Temperate Tree Species

**DOI:** 10.3389/fpls.2022.769551

**Published:** 2022-03-03

**Authors:** Shanshan Yang, Frank J. Sterck, Ute Sass-Klaassen, J. Hans C. Cornelissen, Richard S. P. van Logtestijn, Mariet Hefting, Leo Goudzwaard, Juan Zuo, Lourens Poorter

**Affiliations:** ^1^Forest Ecology and Forest Management Group, Wageningen University and Research, Wageningen, Netherlands; ^2^Department of Ecological Science, Systems Ecology, VU University (Vrije Universiteit) Amsterdam, Amsterdam, Netherlands; ^3^Landscape Ecology, Institute of Environmental Biology, Utrecht University, Utrecht, Netherlands; ^4^Key Laboratory of Aquatic Botany and Watershed Ecology, Wuhan Botanical Garden, Chinese Academy of Sciences, Wuhan, China

**Keywords:** stem economics spectra, plant strategies, trade-offs, physiochemical traits, plant functions

## Abstract

A central paradigm in comparative ecology is that species sort out along a slow-fast resource economy spectrum of plant strategies, but this has been rarely tested for a comprehensive set of stem traits and compartments. We tested how stem traits vary across wood and bark of temperate tree species, whether a slow-fast strategy spectrum exists, and what traits make up this plant strategy spectrum. For 14 temperate tree species, 20 anatomical, chemical, and morphological traits belonging to six key stem functions were measured for three stem compartments (inner wood, outer wood, and bark). The trait variation was explained by major taxa (38%), stem compartments (24%), and species within major taxa (19%). A continuous plant strategy gradient was found across and within taxa, running from hydraulic safe gymnosperms to conductive angiosperms. Both groups showed a second strategy gradient related to chemical defense. Gymnosperms strongly converged in their trait strategies because of their uniform tracheids. Angiosperms strongly diverged because of their different vessel arrangement and tissue types. The bark had higher concentrations of nutrients and phenolics whereas the wood had stronger physical defense. The gymnosperms have a conservative strategy associated with strong hydraulic safety and physical defense, and a narrow, specialized range of trait values, which allow them to grow well in drier and unproductive habitats. The angiosperm species show a wider trait variation in all stem compartments, which makes them successful in marginal- and in mesic, productive habitats. The associations between multiple wood and bark traits collectively define a slow-fast stem strategy spectrum as is seen also for each stem compartment.

## Introduction

The unique feature that sets trees apart from other life forms is their tall and lignified stem. The stem allows trees to grow for decades to centuries to a large size and compete successfully for light with other trees and life forms. The stems provide multiple functions (Sterck et al., 2005; Chave et al., 2009), which can be broadly grouped into transport and storage of resources and assimilates, mechanical support, and defense. These functions are partly delivered by different stem compartments (i.e., sapwood, heartwood, and bark). The sapwood transports water and nutrients from roots to leaves, whereas the heartwood (i.e., inner wood), which is no longer functional in terms of transport but chemically protected against pathogens by accumulation of antifungal substances (e.g., phenols and tannins) (Hillis, 1987; Domec et al., 2005). The bark fulfills multiple functions, such as storage, transportation, photosynthesis, and protection (Franceschi et al., 2005; Rosell et al., 2014; Tuo et al., 2021). The inner bark and outer bark have different traits and show little coordination because they fulfill different functions. The inner bark transports sugars and secondary compounds from leaves to other organs (Steppe et al., 2015) and contains for some species chlorophyll, thus contributing to plant photosynthesis (Pfanz and Aschan, 2001). The outer bark presents a first defense line against insects, drought, and fire (Loram-Lourenco et al., 2020). The large stem trait variation across species indicates that tree species coordinate their traits and functions in different ways (Hacke and Sperry, 2001), with important implications for species performance (Poorter et al., 2010), species distribution within forests and across climatic regions (Sterck et al., 2011, 2014), and species responses to global change (Cornwell et al., 2009).

### Is There a Slow-Fast Stem Spectrum?

One of the central paradigms in comparative plant ecology is that the species sort out along a slow-fast resource economy spectrum of plant strategies (Reich, 2014). This spectrum ranges from species that conserve carbon and nutrient resources and persist in resource-poor unproductive environments, to species that rapidly acquire light, carbon, water, and nutrient resources, grow fast, and thrive in resource-rich productive environments (Díaz et al., 2004; Lambers and Poorter, 2004; Wright et al., 2004; Poorter et al., 2006). This “slow-fast” spectrum has been widely supported by empirical studies, but these studies are strongly biased toward leaf traits (e.g., Wright et al., 2004; Freschet et al., 2012; Kergunteuil et al., 2018) and root traits (e.g., Weemstra et al., 2016; Carmona et al., 2021; Freschet et al., 2021). Fewer studies have analyzed the fast-slow-spectrum for stem traits (Chave et al., 2009). A study on subarctic life forms found that the leaf and stem traits are coupled and form part of an overall plant strategy spectrum (Freschet et al., 2010), whereas studies across tropical tree species (Baraloto et al., 2010) and across woody biomes (Pietsch et al., 2014) found them to be uncoupled. Yet, these studies included only a limited set of stem traits, such as wood density or nutrient concentrations. To our knowledge, no studies have tested the stem economics spectrum using a comprehensive set of anatomical, chemical, and morphological traits for different stem compartments. There is therefore a strong need to further unpack the stem economics spectrum beyond wood density and its associated traits (Chave et al., 2009).

### Stem Traits, Functions, and Significance of Gymnosperms and Angiosperms

Gymnosperms and angiosperms represent an old phylogenetic split with large consequences for stem form and functioning (Feild and Arens, 2007). Angiosperms can transport water efficiently through wide vessels and form thick-walled fibers for mechanical support, while gymnosperms have narrow tracheids that combine both functions (Sperry et al., 2006). Angiosperms generally have higher xylem conductivity because their vessels can achieve much larger dimensions, but gymnosperms can partly compensate for their limited conduit size due to their efficient torus-margo pits (Pittermann et al., 2006; Song et al., 2021). Many studies hypothesize that there is a general trade-off between hydraulic safety and water transport efficiency, but such a trade-off is not universal: only a quarter of the hydraulic studies have supported this trade-off hypothesis (Gleason et al., 2015). For example, no relationship between hydraulic safety and efficiency was found in a study comparing 14 angiosperm and gymnosperm species (Zhang et al., 2020).

Less attention has been paid to the role of parenchyma in ecological strategies. Radial and axial parenchyma are living cells coordinating physiological processes of plants, such as storage of water, non-structural carbohydrates, and production of chemical defense compounds (Morris et al., 2020; Rosell et al., 2021). Angiosperms have in general more parenchyma than gymnosperms, probably because of their different defense strategies; the antifungal substance produced in angiosperm parenchyma cells avoid the spread of fungi, whereas gymnosperm defense relies more on the occlusion of tracheids and resin-producing ducts synthesized by epithelial cells (Morris et al., 2016). Gymnosperms and angiosperms also differ in their bark traits, for example, in gymnosperms, the phloem cells have greater resistance to sap flow than angiosperms (Jensen et al., 2012). Several studies have shown strong-to-weak associations between bark and wood traits (Rosell et al., 2014, 2015, 2021). For example, density and water content of bark and wood were positively correlated across 50 dry and wet tropical species, implying their coordinated functions in water storage and mechanical support (Poorter et al., 2014), whereas no trait correlation was found between bark and wood of 11 temperate tree species (Zuo et al., 2018). Overall, the qualitative differences between angiosperm and gymnosperm species have been well established but the magnitude of these differences, and how that varies with traits is less clear. Similarly, it remains unclear how a comprehensive set of stem traits is coordinated to fulfill the main stem functions, what are the fundamental trade-offs, and whether the same “slow-fast” strategy spectrum can be found across and within these phylogenetic groups.

In this study, we examine how 20 anatomical, chemical, and morphological stem traits vary across a phylogenetically and ecologically diverse set of 14 temperate European tree species. We selected traits that are important for six main stem functions (hydraulic conductivity, hydraulic safety, storage, metabolism, chemical, and physical strength) and measured traits for three stem compartments (inner wood, outer wood, and bark) that have different developmental origins and partly have different functional roles. To minimize the possible confounding ontogenetic or allometric effects of tree size on anatomical traits (Rungwattana and Hietz, 2018; Fajardo et al., 2020), we selected stem sections of similar (25 cm) diameter and controlled statistically for stem length. We addressed the question how stem traits differ among 14 temperate tree species and what trait trade-offs and plant strategies prevail. We expect the large trait variation to be found between taxa and stem compartments, and trait trade-offs determine a stem strategy spectrum running from “slow” species with conservative traits (e.g., high wood density, thicker conduit wall) to “fast” species with acquisitive traits (e.g., high nutrients level, large conduit diameter). Concerning the traits in stem compartments, we expect strong associations between wood and bark traits because they are partially derived from the same vascular cambium (Evert and Eichhorn, 2006), and because they partially fulfill similar functions in terms of water and photosynthates storage and mechanical support (Rosell et al., 2014).

## Materials and Methods

### Study Sites and Species

The tree species were collected from two sites in Netherlands, located in the same temperate climate zone, with an annual rainfall of 700 mm and an annual mean temperature of 10°C: (1) one site is the Hollandse Hout in Flevoland (52.46N, 5.42E). It was reclaimed from the former Zuiderzee in the 1960s, and it is calcareous, moist, and fertile, with a pH close to neutrality and consists of marine clay, remarked as C and (2) the other site is the Schovenhorst forest estate in the Veluwe region (52.25N, 5.63E). It is well-drained and consists of acidic sandy soil, remarked as S. The trees were extracted from monospecific forestry plantations with an average age of 48 years (range 1,911–1,978) as indicated by the tree ages (Table 1). More details about the study sites are given in Cornelissen et al. (2012).

**TABLE 1:** Basic information of 14 studied tree species.

Species	Abbreviation	Collecting sites	Heartwood occurrence	Taxa	Distribution of pores	Age
*Fraxinus excelsior*	Fra.e	C (clay)	No	Angiosperms	Ring-porous	27
*Betula pendula*	Bet.p	C (clay)	No	Angiosperms	Diffuse-porous	38
*Quercus robur*	Que.r_C	C (clay)	Yes	Angiosperms	Ring-porous	33
*Quercus robur*	Que.r_S	S (sandy)	Yes	Angiosperms	Ring-porous	101
*Fagus sylvatica*	Fag.s	C (clay)	No	Angiosperms	Diffuse-porous	32
*Populus* × *canadensis*	Pop.c	C (clay)	No	Angiosperms	Diffuse-porous	19
*Populus tremula*	Pop.t	S (sandy)	No	Angiosperms	Diffuse-porous	45
*Chamaecyparis lawsoniana*	Cha.l	S (sandy)	Yes	Gymnosperms	Tracheids	61
*Thuja plicata*	Thu.p	S (sandy)	Yes	Gymnosperms	Tracheids	36
*Cryptomeria japonica*	Cry.j	S (sandy)	Yes	Gymnosperms	Tracheids	41
*Taxus baccata*	Tax.b	S (sandy)	Yes	Gymnosperms	Tracheids	43
*Picea abies*	Pic.a_C	C (clay)	Yes	Gymnosperms	Tracheids	32
*Picea abies*	Pic.a_S	S (sandy)	Yes	Gymnosperms	Tracheids	34
*Larix kaempferi*	Lar.k	S (sandy)	Yes	Gymnosperms	Tracheids	46
*Pseudotsuga menziesii*	Pse.m	S (sandy)	Yes	Gymnosperms	Tracheids	28
*Abies grandis*	Abi.g	S (sandy)	No	Gymnosperms	Tracheids	42

*For angiosperm Quercus robur (Que.r) and gymnosperm Picea abies (Pic.a), they were extracted from two contrasting sites and labeled as Que.r_C, Que.r_S, Pic.a_C, and Pic.a_S.*

Fourteen species were harvested from these two sites, of which, six were angiosperm species and the other eight were gymnosperm species (Table 1). All these species are common in Europe, and most species occur frequently in Dutch forests, but only six species are native. Because the species differ in their distribution, we collected samples of most angiosperm species from the tree plantations on clay and samples of most gymnosperm species from the tree plantations on sand. There were two exceptions: individuals of the angiosperm *Quercus robur* (Que.r) and gymnosperm *Picea abies* (Pic.a) were extracted from both sites, and labeled them as Que.r_C, Que.r_S, Pic.a_C, and Pic.a_S separately. These two species allowed us to quantify the site effects, and the results confirmed our expectation that site effects on most stem traits were non-significant (Supplementary Table 1).

### Sampling and Variables

The studied tree species were harvested at two different times; ten of them were harvested in February 2012, while the other four were harvested in February 2015. For each tree species, five individual trees were harvested, providing 85 trees. The selected trees were well developed and had an average stem diameter of 25 ± 3 cm. Two adjacent 2-cm chain-sawed disks of each individual tree were collected from the base of the main stem and used for measurements. In this study, we took samples from different stem compartments; the stem consists of wood and bark, and may convert the inward part of the functional sapwood into heartwood, which is no longer functional in terms of transport but chemically protected against pathogens by accumulation of antifungal substances (e.g., phenols and tannins) (Hillis, 1987; Domec et al., 2005).

The wood density was measured based on four 1.5 cm^3^ blocks extracted from inner wood and outer wood of the base disk, respectively. In addition, several 1.5 cm^2^ bark pieces were extracted to measure the bark punch resistance. The wood anatomical traits were measured based on one block of inner wood and outer wood, respectively. The chemical traits were measured based on sawdust samples taken from the disk using an electric drill (bit diameter 8 mm). The plate and drill were cleaned between samples with 70% ethanol; sawdust from inner wood, outer wood, and bark were collected separately for each disk and stored for chemical traits measurement.

In total, 20 traits (Supplementary Table 2) were measured in three compartments (inner wood, outer wood, and bark) of 14 temperate species. Although it is acknowledged that traits have multiple functions, we assigned, for the sake of overview and synthesis, each trait to the stem function to which it contributes the most. The six stem functions are: hydraulic conductivity, hydraulic safety, storage, metabolism, physical defense, and chemical defense. For a definition of the traits and how they are linked to functions, refer Supplementary Table 3. We acknowledge that the inner wood does not contribute to hydraulic conductivity and safety, but included the hydraulic trait values of inner wood to provide a complete and balanced overview of the traits in different stem compartments, and because it allows to evaluate to what extent hydraulic wood traits vary during tree development when trees increase in size, and whether the species ranking is maintained. The comparison of trait coordination between wood and bark is unbalanced, since we did not measure hydraulic traits in bark. We, however, did measure the ten bark traits related to nutrient levels, storage ability, and physiochemical defenses, and those can be compared with similar wood traits. We thus focus the comparison between stem and bark on these nutrition value and defense-related wood-bark associations.

### Physical Traits

#### Wood Density and Heartwood Proportion

The wood density and heartwood proportion were measured on five individual trees for each species. Four blocks were taken along the diameter of disk. The wood density was determined by the water displacement method as mass after drying at 105°C divided by fresh volume. The average density of two innermost blocks represented the inner wood (in some cases heartwood) density, while two outermost blocks represented the outer wood (i.e., sapwood) density. All the disks were assumed to be cylinders, therefore we calculated the heartwood proportion based on the following formula:${\frac{\pi \,r}{\pi \,R}}_{{}^{2}}^{2}$, where r is the width of heartwood and R is the radius of the selected disk. This calculation could only be done for heartwood forming tree species (Table 1).

#### Wood Anatomical Traits

The anatomical traits were measured for three individual trees per species. Transverse sections of wood samples were taken from inner wood and outer wood, and used for anatomical measurements.

To produce permanent samples, the thin sections were prepared in five steps: (1) samples were cut using a sliding microtome (wood samples that were too hard to be cut were first softened in boiling water); (2) a 5% hypochlorite solution was applied to bleach the samples, then the samples were rinsed thoroughly using demi-water; (3) the samples were stained with a mixture of Astrablue and Safranin for at least 5 min; (4) the samples were washed with demi-water and dehydrated in ethanol series (50, 96, and 100%); and (5) thin sections were dewaxed using Roticlear^®^ (Carl Roth, Karlsruhe, Germany) and permanently embedded with Roti^®^-Mount (Carl Roth, Karlsruhe, Germany). High-resolution digital images of anatomical sections were made using a camera mounted on an optical microscope. The gymnosperm sections with narrow tracheids were measured using a lens of 10× magnification, while sections of angiosperms with wider vessels were measured using a lens of 5× magnification. Digital images were calibrated with a slide-mounted micrometer and then analyzed using Fiji/ImageJ (Schindelin et al., 2012), and conduit density, diameter, conduit fraction (i.e., measured as the fraction of cross-sectional area represented by conduits), wall thickness and ray fraction (i.e., measured as the fraction of cross-sectional area represented by rays), fiber wall thickness (for angiosperms only), and the ratio of conduit wall thickness to conduit radius were quantified.

The potential hydraulic conductivity (K*_*p*_*) was calculated according to the Hagen-Poiseuille law (Sterck et al., 2008):


(1) $$K\,p=\left(\pi \,\rho_{w}/128\,\eta \right)\,\text{×}\,C\,D\,\text{×}\,D^{4}$$

where K*_*p*_* is the potential specific stem conductivity (in kg m Mpa^–1^ s^–1^), η is the viscosity of water at 20°C (1.002 × 10^–3^ Pa s at 20°C), ρ_*w*_ is the density of water at 20°C (998.2 kg m^–3^ at 20°C), CD is the conduit density, and D is the conduit diameter (in m).

#### Bark Punch Resistance

The bark punch resistance was used as an indicator of bark toughness. A Mecmesin Ultra Test with AFG-1000N force gauge (Slinfold, West Sussex, United Kingdom) was applied and the bark punch resistance (N) was determined as the maximum force needed to penetrate with a stainless steel needle of 1 mm diameter of the extracted bark pieces. The acceleration was standardized to 150 mm per minute for all samples. The bark toughness may be different along the circumference of the disk, therefore, three to five pieces (1.5 × 1.5 cm) of barks were extracted randomly along the circumference of base disk.

### Chemical Traits

The chemical traits were measured from sawdust samples taken from the inner wood, outer wood, and bark. The samples were ground into fine powder with a Retsch MM400 ball mill (Retsch, Haan, Germany) and oven-dried (48 h at 70°C) to achieve sufficient homogeneity. For samples from the three compartments, we measured the concentrations of carbon, nitrogen, phosphorus, lignin, lignin/cellulose ratio, phenols, and tannins; pH value was measured too, and the C/N ratio was calculated.

The carbon and nitrogen concentrations were determined by dry combustion using a Flash EA 1112 elemental analyzer (Thermo Fisher Scientific, Rodana, Italy).

The phosphorus concentration was determined by digestion with HNO_3_/HCl (1:4 mixture of 37% HCl and 65% HNO_3_). P concentration was determined with spectrophotometry using the ammonium molybdate method (at a wavelength of 880 nm) as proposed by Murphy and Riley (1962).

The lignin and cellulose concentrations were determined following Poorter and Villar (1997). The samples were extracted with water, methanol, and chloroform to remove the soluble sugars, soluble phenols, and lipids. Then starch, fructan, pectin, and a part of the hemi-cellulose were removed during acid hydrolysis. Finally, after correction for ash concentration (including silicates) and remaining proteins, the lignin and cellulose concentrations were calculated based on their difference in C concentration.

The Folin-Ciocalteu method was used to determine the total phenols and tannins concentrations. A 50% methanol solution was used to extract the phenolic hydroxyl groups. Then the phenols were colored with a Folin-Ciocalteu reagent and the samples were measured at 760 nm on a spectrophotometer. Finally, the total phenols were calculated according to a tannic acid-based calibration curve, and the non-tannin phenols were measured after binding the tannins in the 50% methanol extract with polyvinylpolypyrrolidone (PVPP).

The pH_*H*2*O*_ was measured according to Cornelissen et al. (2006). Briefly, 0.15 ml sample was added to 1.2 ml demi-water in an Eppendorf tube and shaken for 1 h at 250 rotations per minute. Then the tubes were centrifuged for 5 min at 13,000 rpm and the supernatant was used to measure pH_*H*2*O*_ value using a narrow (5 mm diameter) SenTix Mic electrode connected to an Inolab Level 2 pH meter (both: Wissenschaftlich-Technische Werkstätten GmbH & Co. KG, Weilheim, Germany).

### Statistical Analysis

Statistical analyses were performed using R v. 3.6.1 (R Core Team, 2016) and CANOCO 5.0 (ter Braak and Smilauer, 2012). All trait variables with the exception of heartwood proportion were log_10_ transformed to increase normality and homoscedasticity.

To evaluate how much trait variation was explained by major taxa (angiosperm vs. gymnosperm), species within major taxa, and stem compartments (inner wood, outer wood, and bark), a variance component analysis was performed using the R package “*variancePartition*” (Hoffman and Schadt, 2016). This package uses a linear mixed model, built on top of the lme4 package (Bates et al., 2015), to partition the variance attributable to multiple variables in the data. The contribution of each variable was expressed as the fraction of explained variation. These categorical variables (major taxa, species nested in major taxa, and stem compartments) are modeled as random effects to obtain statistically valid results as specified in “*variancePartition*” package.

To test which factor(s) significantly contribute to trait variation, a variance partitioning and multiple linear effect model were fitted with each trait as response variable, and major taxa, species within major taxa, and stem compartments as fixed factors. We included stem length (i.e., the estimated vertical distance from stem disk to tree top) as a covariate in these analyses to correct for its potential confounding effects (Supplementary Table 4 and Supplementary Figure 1). Detailed information about stem length calculations is shown in Supplementary Material.

To evaluate how the traits were associated, a principal-component analysis (PCA) was performed using species-mean trait values per compartment type (i.e., inner wood, outer wood, and bark) as data points. In total, 20 stem traits linked to six plant functions were included in the PCAs (for the full list of variables and their functionality, see Supplementary Table 3). To evaluate whether the same trait associations are found within anatomically distinct groups, two additional PCAs were carried out for angiosperm and gymnosperm species separately. The PCA analyses were performed in CANOCO 5.

## Results

### Stem Trait Variation

**FIGURE 1:**
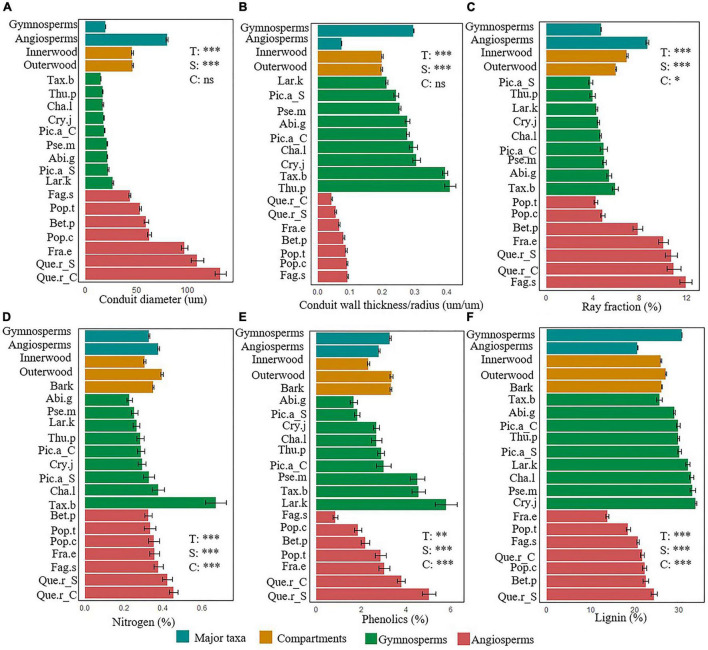
Trait variation between two major taxa (gymnosperms vs. angiosperms; blue-green), across different stem compartments (inner wood, outer wood, and bark; brown) and species nested in major taxa (gymnosperms dark green, angiosperms reddish brown). For each of the six stem functions, one representative trait is shown: **(A)** conduit diameter, **(B)** conduit wall thickness/radius ratio, **(C)** ray fraction, **(D)** nitrogen, **(E)** phenolics, and **(F)** lignin concentrations. Means and SE of the mean are shown. T, Major taxa; S, Species nested within taxa; C, Compartments. ^***^P < 0.001, ^**^P < 0.001, and *P < 0.05, ns, not significant. For species abbreviations see Table 1.

**FIGURE 2:**
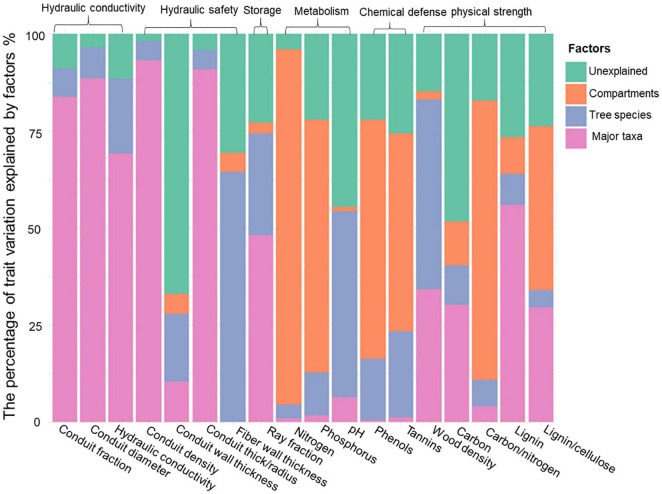
The percentage of trait variation explained by major taxa (angiosperms vs. gymnosperms; pink), species within major taxa (blue), and compartments (inner wood, outer wood and bark, orange), and the unexplained variation (green). The traits are grouped into six stem functions: hydraulic conductivity, hydraulic safety, storage, metabolism, chemical defense, and physical strength.

**FIGURE 3:**
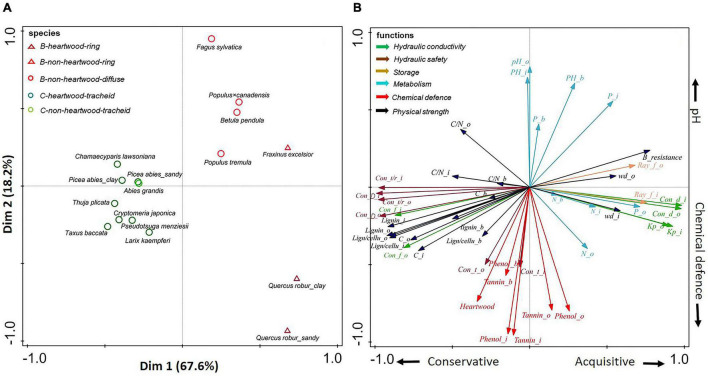
**(A,B)** Principal-component analysis of 20 stem traits of three compartments (inner wood, outer wood, and bark) of all tree species. Dark green circles show gymnosperms with heartwood; light green circles show gymnosperms without heartwood; dark red triangles show ring-porous angiosperm species with heartwood; light red triangles show ring-porous angiosperm species without heartwood; light red circles show diffuse-porous angiosperm species without heartwood. The traits (indicated with arrows) are grouped according to six functions: hydraulic conductivity (in green), hydraulic safety (in dark brown), storage (in light brown), metabolism (in blue), chemical defense (in red), and physical strength (in black). For trait abbreviations see Supplementary Table 2.

**FIGURE 4:**
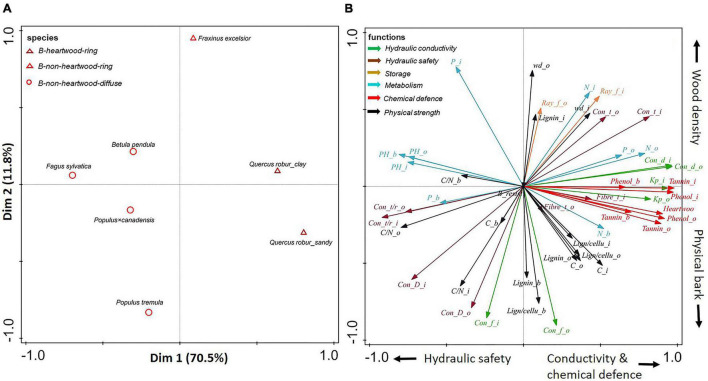
**(A,B)** Principal-component analysis based on stem traits assessed in three compartments (inner wood, outer wood, and bark) for six angiosperms. Dark red triangles show ring-porous angiosperm species with heartwood; light red triangles show ring-porous angiosperm species without heartwood; light red circles show diffuse-porous angiosperm species without heartwood. The traits (indicated with arrows) are grouped according to six functions: hydraulic conductivity (in green), hydraulic safety (in dark brown), storage (in light brown), metabolism (in blue), chemical defense (in red), and physical strength (in black). For trait abbreviations, see Supplementary Table 2.

**FIGURE 5:**
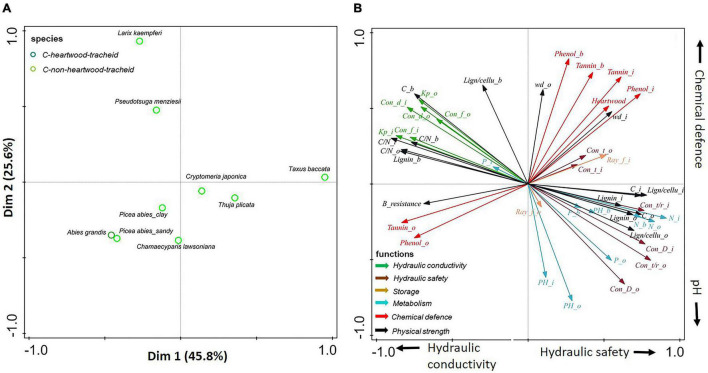
**(A,B)** Principal-component analysis based on stem traits assessed in three compartments (inner wood, outer wood, and bark) for eight gymnosperms. Dark green circles show gymnosperms with heartwood; light green circles show gymnosperms without heartwood. The traits (indicated with arrows) are grouped according to six functions: hydraulic conductivity (in green), hydraulic safety (in dark brown), storage (in light brown), metabolism (in blue), chemical defense (in red), and physical strength (in black). For trait abbreviations, see Supplementary Table 2.

The major taxa (angiosperms vs. gymnosperms), species, and compartments differed in stem traits (Table 2 and Figure 1). All the stem traits (100%) were significantly affected by species and major taxa and ca. 80% by stem compartments. Most of the variation was explained by major taxa (40%), followed by stem compartments (24%) and species within major taxa (19%) (Table 2 and Figure 2). The corresponding values are shown in Table 3. Below, we first discuss the major taxon and compartment effects (Tables 2, 3) whereas species differences are discussed in detail based on the PCA analysis (Figures 3–5).

**TABLE 2:** The percentage of trait variation explained by major taxa (angiosperms vs. gymnosperms), species within major taxa, and compartments (inner wood, outer wood, and bark).

Ecological function	Stem traits	Unit	Major taxa	Species	Compartments
Hydraulic conductivity	Conduit fraction	%	83.7***	7.32***	0.00*^ns^*
	Conduit diameter^†^	μm^2^	88.5***	7.99***	0.04*^ns^*
	theoretical hydraulic Conductivity (*K*_*p*_)^†^	kg m MPa^–1^ s^–1^	69.2***	19.3***	0.09*^ns^*
	**Average**		**80.5**	11.5	0.04
Hydraulic safety	Conduit density^†^	cm^–2^	93.1***	5.09***	0.00*^ns^*
	Conduit wall thickness	μm	10.4**	17.5**	4.98**
	Conduit wall thick/radius^†^	μm/μm	90.9***	4.84***	0.00*^ns^*
	Fiber wall thickness	μm	NA	64.3***	5.11*
	**Average**		**64.8**	22.9	2.52
Storage	Ray fraction	%	**48.2** ***	26.1***	2.71*
Metabolism	Nitrogen^†^	%	0.93***	3.58***	91.4***
	Phosphorus	%	1.61***	11.1***	65.1***
	pH^†^	NA	6.25***	47.9***	1.30*
	**Average**		2.93	20.9	**52.6**
Chemical defense	Phenols^†^	%	0.38**	15.8***	61.6***
	Tannins^†^	%	1.22***	22.2***	51.0***
	**Average**		0.80	19.0	**56.3**
Physical strength	Wood density	g cm^−3^	34.3***	48.8***	2.16**
	Carbon	%	30.1***	10.2***	11.5***
	Carbon/nitrogen	NA	3.90***	7.00***	71.9***
	Lignin	%	55.9***	7.94***	9.59***
	Lignin/cellulose^†^	NA	29.4***	4.55***	42.1***
	**Average**		30.7	15.2	**29.6**
Average			**38.0**	19.3	24.0
*% Significance*			***100***	***100***	***77.2***

The variance explained by the most important factor is given in bold. Asterisks indicate the significance level. At the bottom of the table, the % significant indicates what percentage of measured traits was significantly affected by each factor. ^†^Traits were log_10_-transformed. ***P < 0.001, **P < 0.001, and *P < 0.05.

*ns, not significant.*

**TABLE 3:** Stem trait variation in % among compartments of angiosperm and gymnosperms.

Ecological function	Stem traits	Unit	Angiosperms			Gymnosperms		
			Inner wood	Outer wood	Bark	Inner wood	Outer wood	Bark
Hydraulic conductivity	Conduit fraction	%	17.7	18.7	–	39.3	39.6	–
	Conduit diameter	μm^2^	110	122	–	31.2	33.7	–
	Theoretical hydraulic conductivity (K*p*)	kg m MPa^–1^ s^–1^	62.5	76.9	–	5.00	5.78	–
Hydraulic safety	Conduit density	cm^–2^	69.5	59.8	–	1471	1334	–
	Conduit wall thickness	μm	2.50	2.57	–	2.68	2.88	–
	Conduit wall thick/radius	μm/μm	0.07	0.07	–	0.29	0.29	–
	Fiber wall thickness	μm	2.67	2.83	–	–	–	–
Storage	Ray fraction	%	9.53	7.80	–	4.84	4.54	–
Metabolism	Nitrogen	%	0.09	0.12	0.66	0.07	0.09	0.61
	Phosphorus	%	0.006	0.015	0.05	0.002	0.007	0.04
	pH	NA	5.3	5.11	5.07	4.83	4.95	4.63
Chemical defense	Phenols	%	2.14	0.74	4.43	2.31	0.49	5.49
	Tannins	%	1.84	0.51	2.93	1.73	0.33	3.86
Physical strength	Wood density	g cm^−3^	0.53	0.53	–	0.40	0.44	–
	Carbon	%	46.2	46.0	48.1	48.8	48.4	49.5
	Carbon/nitrogen	NA	550	445	75.1	806	578	93.5
	Lignin	%	16.7	16.7	23.6	29.0	30.0	32.0
	Lignin/cellulose	NA	0.52	0.48	1.26	0.95	0.96	1.44

*Mean trait values of inner wood, outer wood, and bark are shown for angiosperms (N = 6 species) and gymnosperms (N = 8).*

The differences between gymnosperms and angiosperms largely explained the differences in traits related to hydraulic conductivity, hydraulic safety, storage, and physical strength (Table 2). The conduit diameters were on an average threefold larger in angiosperm species than in gymnosperms (Table 3 and Figure 1). Despite the 20-fold larger conduit density in gymnosperms, angiosperm species had a theoretical conductivity that was approximately 12-fold higher compared with gymnosperms due to the strong effect of conduit diameter (Hagen-Poiseuille law, see section “Materials and Methods”). Also, the variation in our best proxy for hydraulic safety (conduit wall thickness to conduit radius ratio) was best explained by major taxon differences. The major taxa also explained most of the variation in ray parenchyma, with angiosperms having a larger ray fraction (∼10%) than gymnosperms (∼5%, Table 3 and Figure 1). Finally, gymnosperms had a higher lignin concentration but a lower wood density (0.42 g cm^–3^) than angiosperms (0.53 g cm^–3^). Remarkably, these two major taxa hardly differed in other physical strength traits, neither in any of the traits related to metabolism or chemical defense.

The stem traits also varied significantly among different tree species (Table 2). Oak (*Quercus robur*) had the largest conduit diameter, high ray fraction, nitrogen, and phenolics concentration (Figure 1), therefore it had strong ability in hydraulic conductivity, nutrient storage, and chemical defense. Of all angiosperm species, Beech (*Fagus sylvatica*) had the largest storage capacity, as indicated by its highest ray fraction (Figure 1C), but the lowest chemical antifungal defense, as indicated by the low concentration of phenols (Figure 1E). Gymnosperms had higher hydraulic safety and physiochemical defenses compared with angiosperms. Among the eight gymnosperm species, *Thuja plicata* had the thickest relative conduit wall thickness (Figure 1B), *Larix kaempferi* had the highest phenolics concentration though its relative conduit wall thickness was smaller compared with other gymnosperm species (Figures 1B,E). *Cryptomeria japonica* had higher physical defense as indicated by its higher lignin concentration, while *Fraxinus excelsior* had the lowest lignin concentration (Figure 1F).

The stem compartments (i.e., inner wood, outer wood, and bark) strongly varied in traits related to metabolism (53%), chemical defense (56%), and, partially, physical strength (30%). For metabolic traits, the N and P concentrations were significantly higher in bark than in both wood components (Table 3 and Figure 1), and higher in the younger outer wood compared with the older inner wood. With respect to chemical defense, bark was best defended by having the highest concentration of phenols and tannins, followed by inner wood, and then outer wood (Table 3). The opposite was true for physical strength; the carbon:nitrogen ratio was higher in both wood compartments compared to bark. The inner wood and outer wood compartments did not vary significant in traits related to hydraulics and storage (bark was not included in these measurements).

We checked for the potential confounding effect of stem length on trait values and found that the stem length had little effect on the traits, given only 0–2.58% trait variation was explained by the stem length (Supplementary Table 4 and Supplementary Figure 1).

### Associations Among Stem Traits

For all species, the first two PCA axes explained almost 86% of the variation in stem traits (Figure 3). Strong major taxon differences determined the variation explained by the first PCA axis, running from gymnosperms with strong physical strength (e.g., lignin) and hydraulic safety (e.g., conduit wall thickness: radius ratio), to angiosperm species with the opposite suite of traits, as well as high conductance traits (e.g., conduit diameter and K*_*p*_*) and large storage capacity (i.e., ray fraction). Perpendicularly, independent of the major taxon effect, an apparent trade-off was found between chemical defenses (e.g., phenols) and pH, which at least partially ran parallel for both gymnosperms and angiosperms.

A large trait variation was observed within angiosperms; ring-porous oak (*Quercus robur*) and ash (*Fraxinus excelsior*) had stronger hydraulic conductivity (larger conduit diameter) and higher wood density, while diffuse-porous birch (*Betula pendula*), beech (*Fagus sylvatica*), and both poplar species (*Populus tremula* and *Populus* × *canadensis*) were clustered with higher pH values (with an average of 5.5) (Supplementary Table 5). In contrast to the large trait variation within angiosperm species, there was convergence of trait variation within gymnosperms. Subsequently separate PCAs (Figures 4, 5) for angiosperm and gymnosperm species, to remove the dominant major taxon effect, showed rather distinct trait associations within these two major taxa.

For angiosperms only, the first two axes of the PCA explained ca. 82% of the variation in stem traits (Figure 4). The first PCA axis explained 70.5% of the trait variation, running from beech (*Fagus sylvatica*) with small conduits and high pH through the other diffuse-porous species to ring-porous oak with heartwood. Hence, this first axis ran from relatively high hydraulic safety (e.g., relatively thicker vessel walls) and low conductance to wood that was more conductive and better chemically defended by heartwood formation. The second axis ran from *Populus tremula* with strong physically defended bark and weaker defended wood, to ring-porous ash (*Fraxinus excelsior*) with relatively strong physical strength (e.g., high wood density) to compensate for a lack of chemical defense.

For gymnosperms only, the first two axes explained 46% of the trait variation (Figure 5). The first PCA axis indicates a phylogenetic split between the Pinaceae (*Larix kaempferi*, *Pseudotsuga menziesii*, and *Picea abies*) vs. the Cupressaceae (*Chamaecyparis lawsoniana*, *Cryptomeria japonica*, and *Thuja plicata*) together with the closely phylogenetically related yew (*Taxus baccata*) from the Taxaceae. Accordingly, we found that this axis ran from higher hydraulic conductivity (e.g., conduit diameter and K*_*p*_*) to higher hydraulic safety (e.g., thicker conduit walls), nutrient concentrations (i.e., N and P), and storage ability (i.e., ray fraction). The second axis was mainly driven by bark chemical defense and pH, running from *Chamaecyparis lawsoniana* with high pH to *Larix kaempferi* and *Pseudotsuga menziesii* with denser and better chemically protected bark.

The associations between multiple wood and bark traits collectively define a stem strategy spectrum, ranging from a conservative to an acquisitive strategy. Such a strategy spectrum can be found in wood and bark compartments, with conservative traits associated with physical defense and acquisitive traits associated with high nutrient concentrations and metabolic activity (Figure 3). Pearson correlations were conducted to evaluate trait associations among different stem compartments (Supplementary Figures 2–4). Most of the traits are significantly coordinated or uncorrelated between inner wood and outer wood when all tree species or each tree major taxon were included. However, when only looked at gymnosperms, a negative correlation was found between inner wood and outer wood in terms of phenolics (with Pearson correlation coefficient *r* = −0.66) and tannins (*r* = −0.60) (Figure 5 and Supplementary Figure 4). Moreover, wood and bark were uncoupled for most traits (*P* > 0.05), with the exception of lignin concentration and C/N in wood that were positively correlated with those in bark (*P* > 0.05, Supplementary Figure 2).

## Discussion

We evaluated the following: (1) how do stem traits differ among major taxa (gymnosperm vs. angiosperm), species within major taxa, and stem compartments and (2) how traits are coordinated and/or traded-off against one another. As expected, most of the trait variation was explained by major taxa (38%), followed by compartments (24%) and species within major taxa (19%). The stem trait variation in temperate tree species is therefore mainly explained by an old phylogenetic split, which has led to a fundamentally different anatomy and characteristics of wood compartments between gymnosperms (with narrow tracheids, lack of fibers, but higher hydraulic safety and chemically protected heartwood formed in the majority of species) and angiosperms (with wide vessels embedded in fibers enabling high conductivity and with lack of chemically protected heartwood in most, i.e., diffuse porous species). The trade-offs between hydraulic traits were less marked between angiosperms species and specially between gymnosperm species. Angiosperm species nevertheless largely diverged in their trait values, whereas gymnosperms tended to converge in their trait values. As expected, the stem compartments differ in traits with bark serving as storage organ and a first defense layer (Srivastava, 1964; Romero et al., 2009; Rosell et al., 2014), whereas the wood components were protected by physical and chemical (heartwood) defense. In the discussion, we will first focus on trait variation and associations between major taxa, then within each major taxon, and finally discuss coordination across stem compartments.

### Conservative Gymnosperm vs. Acquisitive Angiosperm Species

We first asked how stem traits vary between gymnosperm and angiosperm species. Thirty-eight percent of the trait variation was explained by these two taxa, particularly for traits related to hydraulic conductivity, hydraulic safety, and storage, but less for chemical traits (Table 2). A multivariate trait analysis of all species combined (Figure 3) confirmed that the trait variation was largely driven by major taxon differences and reflected possible coordination and trade-offs among stem traits.

The angiosperms achieved a 10 times higher theoretical hydraulic conductivity than gymnosperms (Table 3) because they had ca. four times wider conduit diameters (Tyree and Zimmermann, 2002). Instead, the gymnosperm species had a higher resource investment in hydraulic safety, as they had ca. fourfold higher ratio of conduit wall thickness to conduit radius, and higher conduit density compared with angiosperm species (Table 3 and Figure 1). A strong trade-off between high hydraulic safety of gymnosperms vs. high hydraulic conductivity and wood density of angiosperms was found in the multivariate analysis (Figure 3). This finding implied that the reinforced conduits of gymnosperms can better resist implosion when drought causes strong water tension (Pittermann et al., 2006; Blackman et al., 2010), while the high water conductivity of angiosperms enable to sustain high transpiration rates of their thin leaves, attain high photosynthetic rates, and become successful also in mesic, productive habitats. This high productivity allows these winter-deciduous angiosperms to partly compensate for their shorter growing season (Reich et al., 1997).

The angiosperms had a twofold higher ray fraction compared with gymnosperms (Table 3 and Figure 1) (cf. Morris et al., 2016; Zhang et al., 2020), indicating a larger capacity for carbohydrate, water, and nutrient storage. Ray parenchyma has multiple functions as it contributes to radial strength (Reiterer et al., 2002), defense against pathogens (Jupa et al., 2016), transition of sapwood into heartwood (Spicer and Holbrook, 2007), and storage of non-structural carbohydrates (Rosell et al., 2021) that allow for cell metabolism and cavitation repair, and can also fuel new growth of trees (Rosas et al., 2013). The winter-deciduous angiosperm species may require a high carbohydrate storage capacity to survive winter and flush new leaves in spring (Epron et al., 2012), whereas the evergreen gymnosperms can flush new leaves later in spring using photosynthates coming from their preserved needles rather than stored carbohydrates (Lusk, 2001). The parenchyma cells in angiosperms are also important to produce a large amount of extractives to avoid the spread of decay (Sun et al., 2007; Morris et al., 2016), whereas gymnosperm defense relies more on the occlusion of tracheids and abundant antifungal compounds produced by resin ducts (Hudgins et al., 2004; Fuhr et al., 2013).

Interestingly, most angiosperms physically protected their wood by having a 1.3-fold higher wood density (cf. Barnett and Jeronimidis, 2003), mainly realized through thick-walled fibers than gymnosperms, whereas gymnosperms protect their wood by having a twofold higher lignin concentration than angiosperm species (cf. Lamlom and Savidge, 2003). In addition, the lignin of gymnosperms has more condensed carbon-carbon inter-unit bonds that can enhance wood stiffness (Lourenço et al., 2015). Moreover, a larger trait variation and a range of strategies were observed for angiosperm species (cf. Olson et al., 2020), whereas convergence of trait values and more similar strategies were found for gymnosperm species. This larger variation in stem traits is also paralleled by a larger variation in leaf and crown traits (Wright et al., 2004), where leaves of angiosperm species vary largely in size and shape, whereas gymnosperms make small needles and scale leaves, have mostly an evergreen leaf habit, and they make regular horizontal branches that are under stronger apical control.

In summary, gymnosperms showed strong convergence in their traits and have many narrow reinforced tracheids that increase hydraulic safety and high lignin concentrations that increase physical defense. Such a “slow and safe” strategy may allow gymnosperms to avoid freezing- and drought-induced cavitation, have persistent evergreen leaves, and occupy dry, unproductive habitats. Angiosperms are more diverse in stem traits and combine high hydraulic conductivity with storage parenchyma. Paradoxically, angiosperms also have a high wood density, as they have (relatively heavy) fibers that are needed to strengthen the larger conduits. Such a “fast and efficient” strategy allows them to be winter deciduous and occupy a large variety of habitats including mesic, seasonally productive habitats.

### Angiosperm Species Show a Trade-Off Between Diffuse and Ring-Porous Species

Within angiosperm species, the diffuse porous trees have higher hydraulic safety (e.g., narrow vessels and relatively thicker vessel walls), which allows them to avoid freezing-induced cavitation. Therefore, these tree species are able to flush earlier in springtime with the benefit of a longer growing season. In contrast, ring-porous species avoid the risk of freezing-induced cavitation by flushing later, but the shorter growing season is compensated by the higher hydraulic conductivity and chemical defense due to their wider vessels and higher phenols concentrations.

The ring-porous ash had a higher wood density compared with diffuse-porous poplar (Figure 4) (cf. Zobel and Van Buijtenen, 2012), probably because the large vessels of ring-porous species are embedded in a matrix of thick-walled fibers that increase wood density (Zobel and Van Buijtenen, 2012). The lower wood density of poplar indicated its weaker wood defense, but poplar may compensate for its weakly defended wood by increasing its bark physical defense as indicated by the higher bark lignin concentration and thicker bark (Yang et al., 2021). Oak also stood out in chemical defense by high phenols and tannin concentrations in its heartwood, which contributes to its longevity.

In summary, simple qualitative anatomical features such as vessel arrangement and size (diffuse- vs. ring-porous wood) are associated with as much as 71% of the quantitative variation in anatomical, chemical, and morphological traits (Figure 4).

### Gymnosperms Show a Trade-Off Between Efficient Pinaceae and Safe Cupressaceae

For gymnosperms, as expected, a strong trade-off was found between hydraulic conductivity and hydraulic safety since both functions are determined by their tracheids in gymnosperms, while angiosperm trees use vessels functioning in conductivity and fibers, respectively, functioning in mechanical support (Zanne et al., 2010). Specifically, Cupressaceae produce more tracheids and thicker walls that increase hydraulic safety, whereas Pinaceae produce wider tracheids that increase conductivity, growth, and productivity (Cochard et al., 2004; Pittermann et al., 2006). Counterintuitively, the Cupressaceae (*Chamaecyparis lawsoniana*, *Cryptomeria japonica*, and *Thuja plicata*) in our study tend to come from warm and wet areas, where they are known as fast growing species. Such high growth rates may be facilitated by high metabolic rates, as indicated by the high stem nutrient concentrations. Gymnosperms showed coordinated hydraulic conductivity and physical strength (Figure 5), as they combined high hydraulic conductivity with well defended bark (e.g., high bark resistance and lignin concentration). This indicated that gymnosperms, especially the Pinaceae (e.g., *Pseudotsuga menziesii*, and *Larix kaempferi*), can be both productive and well defended.

In summary, strategy variation in gymnosperms is determined by a phylogenetic split, which generally runs from Cupressaceae (*Chamaecyparis lawsoniana*, *Cryptomeria japonica*, and *Thuja plicata*) with high hydraulic safety, metabolic rate, and storage capacity, to Pinaceae (e.g., *Pseudotsuga menziesii*, *Larix kaempferi*) with efficient water transport and well-defended bark.

### Trait Variation and Associations Among Stem Compartments

The stem compartments showed a distinct radial allocation pattern in traits related to metabolism and defense. An average of fivefold higher nitrogen and phosphorus concentrations in bark compared with wood tissues of the studied species are consistent with the literature (cf. Harmon et al., 1986; Adler et al., 2005), and reflect that bark of some tree species may be photosynthetically active and plays an important role in assimilating transport and nutrient storage (Heilman and Stettler, 1986). The fourfold higher phenol and tannin concentrations in the bark compared with wood were consistent with earlier findings that indicated bark can serve as the first defense layer, as phenols and tannins can hamper fungal growth and insect attack (cf. Fengel and Wegener, 2011; Navarro Hoyos et al., 2015; Kahl et al., 2017). Significantly higher phenol concentrations were found in the inner wood (2.2%) compared with the outer wood (0.6%) (cf. Morais and Pereira, 2012), which is due to heartwood production in all but one (*Abies grandis*) of the gymnosperm species and *Quercus robur* among the angiosperm species.

When all the tree species were included, inner wood and outer wood were coordinated or uncoupled for most traits. While for angiosperm species, the chemical defenses of all three compartments were coordinated, for gymnosperm species, a trade-off was found between the chemical defense traits of inner wood and outer wood, probably because most of our gymnosperm species produce heartwood (Hillis, 1987; Domec et al., 2005) whereas for our angiosperm species, only oak produces heartwood. Gymnosperms showed a trade-off between outer wood and bark in terms of chemical defense, indicating that outer wood may invest more resources in other functions (e.g., water and nutrients transportation) when bark and inner wood are well chemically defended. Moreover, lignin and C/N in wood were positively associated with those in bark though most traits varied independently between the two compartments. This trait coordination indicated functional coordination (i.e., physical defense) and is consistent with the previous studies showing that wood and bark traits (i.e., density) were positively associated (Poorter et al., 2014; Rosell et al., 2014).

## Conclusion

The gymnosperms and angiosperms are dominant taxa in temperate and boreal forests. Both across and within these taxa, the associations between multiple wood and bark traits indicate that a conservative-acquisitive stem strategy spectrum is found (cf. Li et al., 2021). A stem trait spectrum is also found for each stem compartment. This spectrum runs from slow hydraulically safe gymnosperms to more diverse, but potentially also fast and hydraulically efficient angiosperms. This indicates that abiotic conditions (drought, freezing) can present a major selective force to trees. Both groups showed a second strategy gradient related to chemical defense, indicating that defense against biotic pests is universally important. Gymnosperms showed strong convergence in their trait strategies because of their uniform tracheids, explaining why they may have a more similar ecological behavior compared with angiosperms.

The stem traits differed significantly among stem compartments; bark had higher concentrations of nutrients and phenolics, whereas sapwood has stronger physical defense as indicated by high carbon to nitrogen ratio. The inner wood of specific conifers and some ring-porous species can be additionally strengthened chemically by heartwood formation. This supports that stem compartments reflected different strategies; bark served as storage organ and a first physical and chemical defense layer, while wood was well physically defended by having stronger tissues.

Yet, most of the traits were uncoupled between wood and bark (*P* > 0.05), indicating the different ways by which the species optimize growth and defense strategies. But co-variation of bark and wood in terms of lignin and C/N indicated the coordinated physical defense function between the two compartments. The diverse trait associations between tree species and stem compartments underpin the diverse plant strategies that have evolved in response to environmental variation. Our generalizations are based on many (20) wood and bark traits of important temperate tree species, but we did not measure the inner and outer barks separately, which can perform entirely different functions (Rosell et al., 2014). It should also be noted that in our study, the extent of the conclusions is limited to two temperate sites (at one site tree came from a forest or a different plantations). Thus, to further unpack the global stem economics spectrum, we recommend that future studies should consider diverse stem compartments (i.e., inner wood and outer wood and bark), diverse tree species, and broad climatic gradients.

## Data Availability Statement

The original contributions presented in the study are included in the article/Supplementary Material, further inquiries can be directed to the corresponding author/s.

## Author Contributions

SY, LP, FS, US-K, and JC conceived the ideas and designed methodology. SY, LP, FS, and US-K analyzed the data. SY led the writing of the manuscript. All authors made contributed to the data collection and critically to the drafts and gave final approval for publication.

## Conflict of Interest

The authors declare that the research was conducted in the absence of any commercial or financial relationships that could be construed as a potential conflict of interest.

## Publisher’s Note

All claims expressed in this article are solely those of the authors and do not necessarily represent those of their affiliated organizations, or those of the publisher, the editors and the reviewers. Any product that may be evaluated in this article, or claim that may be made by its manufacturer, is not guaranteed or endorsed by the publisher.
