# Supplementary material for: Stem Trait Spectra Underpin Multiple Functions of Temperate Tree Species
Source: Front Plant Sci. 2022 Mar 3;13:769551. doi: 10.3389/fpls.2022.769551 (PMC8930200; doi:10.3389/fpls.2022.769551)
Supplement: Supplementary file 1 [file Data_Sheet_1.doc]

Supplementary Material

**Stem Trait Spectra Underpin Multiple Functions of Temperate Tree Species**

# **Methods**

**Testing for possible confounding site effects**

Since only *Quercus robur* and *Picea abies* occurred in both sites (Flevoland and Schovenhorst), site effects could only be tested directly for these 2 species. A two-way ANOVA was conducted to show how species and site contribute to stem trait variation; among the 18 stem traits, only two (conduit fraction and carbon concentration) were significantly, but only marginally, affected by site (Table 1).

Furthermore, for each stem trait, group differences were calculated using the formula (angiosperm-gymnosperm)/(angiosperm+gymnosperm)*100% and site differences using the formula (clay-sand)/(clay+sand)*100%, where the terms angiosperm, gymnosperm, clay and sand refer to averaged traits values for species group or site. For all stem traits, the difference between major taxa outweighed site-specific differences, thus confirming that sites hardly affected stem trait variation. This analysis thus indirectly support our claims on the effect of major taxa effects on traits variation, irrespective of the confounding (but inferior) site effect.

**Estimating xylem conductive length**

The xylem conductive length between the sampled stem disk and the top of the tree may potentially influence the variation in xylem anatomical traits, therefore we incorporate stem length (i.e. tree height above the disk sampling position) as co-variate in our analysis. Given that individual tree height was not measured at the beginning of the experiment, we estimated the tree length for all trees using a step wise approach. We illustrate this approach with results for *Betula pendula* as example:

**St****ep 1: tree hei****ght-DBH curve**


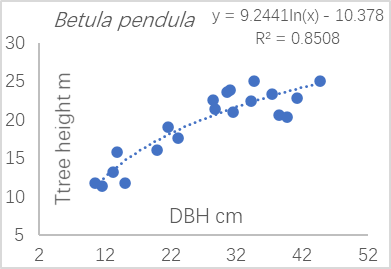
We measured the height and diameter at 1.3 m (DBH) of 10-33 individuals per tree species in their site and fit the tree height and DBH relationship using the equation:

y=9.2441 ln(x)-10.378,

where y is tree height and x is the DBH.

**Step 2: DBH of our sampling individuals**


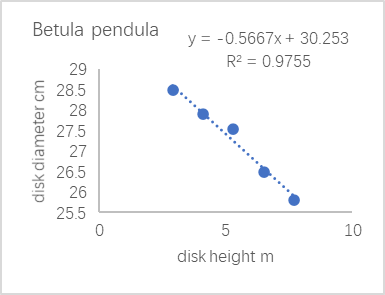
The diameter of disks used in our study is known but we sampled these disks at different height or vertical distance from the soil, ranging from 0 to 10.43 m, in order to get disks of similar diameter. To test for possible stem tapering over this distance, we took advantage of LOGLIFE project in which five logs (A-E) each of 1 m length were sawn from the main trunk. Adjacent to each log, a 2-cm thick disk was sawn out for other analyses in LOGLIFE project (Cornelissen et al., 2012). The diameter and height of the disks were known and in total five disks were used for the diameter tapering rate calculation,

using equation:

y’ = -0.5667x’ + 30.253,

where y’ is the disk height and x is the disk diameter. This allows us the estimate the stem diameter at 1.3 m (DBH), which is 29.5 cm in the shown example.

**Step 3: tree height of our sampling individuals**

Given the DBH obtained from step 2, the height of each individual tree was calculated based on formula 1. As the example shown above: with DBH =29.5 cm, the tree height of that *Betula pendula* tree is estimated 20.9 m.

**Step 4: stem length (xylem conductive length)**

The height above the base disk (used in our study) is considered a more important predictor for stem trait variation than tree height, since it indicates the conductive length between sample disk and tree top. We therefore used this length as the co-variate in our analysis, calculated from the difference between total tree height minus the vertical distance from soil surface to the sampled disk height.

# **Tables and figures**

**Supplementary Table 1.** Trait variation between major taxa (angiosperms vs gymnosperms) and two tree growing sites. Group differences are shown as percentage (%). Two-way ANOVA was applied to test if species, growing sites conditions and interaction between species and sites can significantly contribute to trait variation. Asterisks indicate the significance level of each factor. ***: P<0.001, **: P<0.001, *: P<0.05, ns: not significant.

| **Ecological function** | **Stem traits** | **Angiosperms** | **Gymnosperms** | **Group difference%** | **Significance test**  **(two-way ANOVA)** | | | **Site difference%** |
| --- | --- | --- | --- | --- | --- | --- | --- | --- |
|  |  |  |  |  | **species** | **sites** | **Species*sites** |  |
| **Hydraulic**  **conductivity** | Conduit fraction | 18.42 | 39.46 | 36.35 | *** | ***** | 0.64 | 7.31 |
|  | Conduit diameter | 79.53 | 19.70 | 60.29 | *** | 0.40 | 0.21 | 6.66 |
|  | theoretical hydraulic Conductivity (*K*_p_) | 73.21 | 5.39 | 86.28 | *** | 0.64 | 0.26 | 14.65 |
| **Hydraulic safety** | Conduit density | 64.17 | 1402.28 | 91.25 | *** | 0.27 | * | 12.25 |
|  | Conduit wall thickness | 2.54 | 2.78 | 4.44 | 0.41 | 0.44 | 0.74 | 1.38 |
|  | Conduit wall thick/radius | 0.07 | 0.30 | 60.33 | *** | 0.69 | 0.08 | 3.70 |
|  | Fiber wall thickness | 2.74 | NA | NA | NA | 0.18 | NA | NA |
| **Storage** | Ray fraction | 8.64 | 4.69 | 29.61 | *** | 0.54 | 0.67 | 4.35 |
| **Metabolism** | Nitrogen | 0.37 | 0.33 | 6.84 | * | 0.77 | 0.79 | 5.46 |
|  | Phosphorus | 0.03 | 0.02 | 13.19 | 0.34 | 0.94 | 0.16 | 0.90 |
|  | pH | 5.10 | 4.75 | 3.54 | *** | 0.08 | 0.93 | 2.15 |
| **Chemical defence** | Phenols | 2.79 | 3.28 | 8.06 | * | 0.96 | 0.50 | 0.46 |
|  | Tannins | 1.98 | 2.34 | 8.35 | * | 0.90 | 0.52 | 4.39 |
| **Physical strength** | Wood density | 0.55 | 0.44 | 11.84 | *** | 0.80 | 0.72 | 0.61 |
|  | Carbon | 47.04 | 49.07 | 2.11 | ** | ****** | ** | 1.15 |
|  | Carbon/nitrogen | 286.31 | 417.99 | 18.70 | ** | 0.45 | 0.45 | 2.20 |
|  | Lignin | 20.57 | 30.71 | 19.78 | *** | 0.39 | 0.51 | 2.82 |
|  | Lignin/cellulose | 0.87 | 1.20 | 15.70 | * | 0.68 | 0.52 | 3.00 |

**Supplementary Table 2.** Basic information of measured stem traits. Ecological functions, functional traits, abbreviation, measured compartments, and unit are shown.

| **Ecological functions** | **Functional traits** | **Abbreviation** | **Measured tissues** | **Unit** |
| --- | --- | --- | --- | --- |
| **Hydraulic efficiency** | Conduit fraction | Con_f_i | Inner wood | % |
|  |  | Con_f_o | Outer wood | % |
|  | Conduit diameter | Con_d_i | Inner wood | µm^2^ |
|  |  | Con_d_o | Outer wood | µm^3^ |
|  | Theoretical hydraulic conductivity | K*p*_i | Inner wood | kg m Mpa**^-1^**s**^-1^** |
|  |  | K*p*_o | Outer wood | kg m Mpa**^-1^**s**^-2^** |
| **Hydraulic safety** | Conduit density | Con_D_i | Inner wood | cm^-2^ |
|  |  | Con_D_o | Outer wood | cm^-2^ |
|  | Conduit wall thick | Con_t_i | Inner wood | µm |
|  |  | Con_t_o | Outer wood | µm |
|  | Conduit wall thick/radius | Con_t/r_i | Inner wood | µm/µm |
|  |  | Con_t/r_o | Outer wood | µm/µm |
|  | Fibre wall thick | Fibre_t_i | Inner wood | µm |
|  |  | Fibre_t_o | Outer wood | µm |
| **Storage** | Ray fraction | Ray_f_i | Inner wood | % |
|  |  | Ray_f_o | Outer wood | % |
| **Metabolism** | Nitrogen fraction | N_i | Inner wood | % |
|  |  | N_o | Outer wood | % |
|  |  | N_B | Bark | % |
|  | Phosphorus fraction | P_i | Inner wood | % |
|  |  | P_o | Outer wood | % |
|  |  | P_b | Bark | % |
|  | pH | pH_i | Inner wood | NA |
|  |  | pH_o | Outer wood | NA |
|  |  | pH_b | Bark | NA |
| **chemical defence** | Phenols | Phenol_i | Inner wood | % |
|  |  | Phenol_o | Outer wood | % |
|  |  | Phenol_b | Bark | % |
|  | Tannins fraction | Tannin_i | Inner wood | % |
|  |  | Tannin_o | Outer wood | % |
|  |  | Tannin_b | Bark | % |
|  | Heartwood proportion | Heartwood | Disk | % |
| **physical strength** | Wood density | Wd_i | Inner wood | g/cm^3^ |
|  |  | Wd_o | Outer wood | g/cm^3^ |
|  | Carbon concentration | C_i | Inner wood | % |
|  |  | C_o | Outer wood | % |
|  |  | C_b | Bark | % |
|  | Carbon/nitrogen | C/N_i | Inner wood | NA |
|  |  | C/N_o | Outer wood | NA |
|  |  | C/N_b | Bark | NA |
|  | Lignin fraction | Lignin_i | Inner wood | % |
|  |  | Lignin_o | Outer wood | % |
|  |  | Lignin_b | Bark | % |
|  | Lignin/cellulose | Lign/cellu_i | Inner wood | NA |
|  |  | Lign/cellu_o | Outer wood | NA |
|  |  | Lign/cellu_b | Bark | NA |
|  | Bark punch resistance | B_resistance | Bark | N |

**Supplementary Table 3.** Stem trait definition and related stem functions. Name of stem traits, definition of traits, ecological functions, and supporting literature are listed. Note: one trait may link to several functions, but we only group it to specific function, which we think is most related and important in this study.

| **Stem traits** | **Definition** |  | **Ecological function** | **Supporting literature** |
| --- | --- | --- | --- | --- |
| Conduit fraction | Cross-sectional area of all conduit lumen divided by the total cross-sectional area |  | Hydraulic  conductivity | Dimond, 1966; Chave *et al*., 2009; Martínez-Cabrera, and Estrada-Ruiz, 2014 |
| Conduit diameter | Conduits were assumed to be round and the diameter was calculated based on conduit area measured in ImageJ. |
| theoretical hydraulic Conductivity (*K*_p_) | K*p* was calculated according to the Hagen-Poiseuille law (Sterck *et al*. 2008), see “Materials and Methods” |
| Conduit density | The number of conduits per cross-sectional area |  | Hydraulic safety | Jacobsen, 2005; Chave *et al*, 2009; Janssen *et al*., 2020 |
| Conduit wall thickness | Average wall thickness was measured based on all vessels and multiple tracheids per individual species |
| Conduit wall thick/radius | Conduit wall thickness divided by conduit radius |
| Fiber wall thickness | Fibre wall thickness was measured only in angiosperm species |
| Ray fraction | Cross-sectional area of all ray parenchyma divided by the total cross-sectional area |  | Storage | Morris, 2016 |
| Nitrogen | Mass-based nitrogen (N) concentration |  | Metabolism | Rektorschek *et al.*, 1998; Zhong *et al*., 2017 |
| Phosphorus | Mass-based Phosphorous (P) concentration |
| pH | pH value in distilled  water, pH_H2O_ |
| Phenols | Mass-based phenolics concentration |  | Chemical defence | Mounguengui, 2016; Valette, 2017 |
| Tannins | Mass-based tannins concentration |  |
| Wood density | Oven dry mass divided by fresh volume |  | Physical strength | Zanne *et al*., 2010; Ishida *et al.*, 2008; Sattler and Funnell-Harris, 2013; Wainhouse *et al*., 1990; Rasmann *et al.*, 2011 |
| Carbon | Mass-based carbon (C) concentration |
| Carbon/nitrogen | Carbon concentration divided by nitrogen concentration |
| Lignin | Mass-based lignin concentration |
| Lignin/cellulose | Lignin concentration divided by cellulose concentration |

**Supplementary Table 4.** Percentage trait variation explained by major taxa (angiosperms vs gymnosperms), species within major taxa compartments (innerwood, outerwood, bark) and stem length (between sampled disk and tree top). Asterisks indicate significance levels. †: traits were log-transformed. ***: P<0.001, **: P<0.001, *: P<0.05, ns: not significant.

| **Ecological function** | **Stem traits** | **Unit** | **Major taxa** | **Species** | **Compartments** | **Length** |
| --- | --- | --- | --- | --- | --- | --- |
| **Hydraulic**  **conductivity** | Conduit fraction | % | 83.7*** | 7.32*** | 0.00^ns^ | ns |
|  | Conduit diameter† | µm^2^ | 88.5*** | 7.99*** | 0.04^ns^ | 0.25(+)*** |
|  | theoretical hydraulic Conductivity (*K*_p_)† | kg m MPa^-1^s^-1^ | 69.2*** | 19.3*** | 0.09^ns^ | ns |
| **Hydraulic safety** | Conduit density† | cm^-2^ | 93.1*** | 5.09*** | 0.00^ns^ | 0.20(-)*** |
|  | Conduit wall thickness | µm | 10.4** | 17.5** | 4.98** | ns |
|  | Conduit wall thick/radius† | µm/µm | 90.9*** | 4.84*** | 0.00^ns^ | 0.44(-)*** |
|  | Fiber wall thickness | µm | NA | 64.3*** | 5.11* | ns |
| **Storage** | Ray fraction | % | 48.2*** | 26.1*** | 2.71* | ns |
| **Metabolism** | Nitrogen† | % | 0.93*** | 3.58*** | 91.4*** | 0.44(-)*** |
|  | Phosphorus | % | 1.61*** | 11.1*** | 65.1*** | 2.58(-)*** |
|  | pH† | NA | 6.25*** | 47.9*** | 1.30* | ns |
| **Chemical defence** | Phenols † | % | 0.38^**^ | 15.8*** | 61.6*** | 0.33(-)** |
|  | Tannins† | % | 1.22^***^ | 22.2*** | 51.0*** | 0.04(+)** |
| **Physical strength** | Wood density | % | 34.3*** | 48.8*** | 2.16** | ns |
|  | Carbon | % | 30.1*** | 10.2*** | 11.5*** | ns |
|  | Carbon/nitrogen | NA | 3.90*** | 7.00*** | 71.9*** | 0.52(+)*** |
|  | Lignin | % | 55.9*** | 7.94*** | 9.59*** | ns |
|  | Lignin/cellulose† | NA | 29.4*** | 4.55*** | 42.1*** | 0.22(+)** |
| ***% significance*** |  |  | ***100*** | ***100*** | ***72.2*** | ***50.0*** |

| **Compartment** | **Stem traits** | **Fra.e** | **Bet.p** | **Que.r_C** | **Que.r_S** | **Fag.s** | **Pop.c** | **Pop.t** | **Cha.l** | **Thu.p** | **Cry.j** | **Tax.b** | **Pic.a_C** | **Pic.a_S** | **Lar.k** | **Pse.m** | **Abi.g** |
| --- | --- | --- | --- | --- | --- | --- | --- | --- | --- | --- | --- | --- | --- | --- | --- | --- | --- |
| Wood | Con_f | 8.02 | 18.5 | 16.1 | 23.4 | 17.4 | 20.8 | 23.1 | 38.7 | 36.9 | 37.8 | 32.1 | 39.6 | 43.1 | 47.4 | 40.4 | 39.1 |
|  | Con_d | 96.7 | 59.3 | 132 | 109 | 43.5 | 62.5 | 53.6 | 17.0 | 16.8 | 18.0 | 15.1 | 18.6 | 22.4 | 26.9 | 21.2 | 21.3 |
|  | *K*_p_ | 30.7 | 22.2 | 208 | 169 | 10.8 | 26.1 | 21.5 | 4.0 | 3.5 | 4.3 | 2.4 | 4.4 | 7.2 | 11.0 | 5.7 | 5.9 |
|  | Con_D | 11.0 | 72.9 | 26.8 | 46.4 | 116 | 75.7 | 104 | 1824 | 1639 | 1587 | 1805 | 1478 | 1127 | 874 | 1168 | 1119 |
|  | Cond_t | 3.09 | 2.49 | 2.66 | 2.72 | 1.99 | 2.44 | 2.39 | 2.39 | 3.34 | 2.69 | 2.95 | 2.56 | 2.67 | 2.83 | 2.67 | 2.89 |
|  | Cond_t/r | 0.06 | 0.09 | 0.04 | 0.05 | 0.09 | 0.08 | 0.09 | 0.27 | 0.40 | 0.30 | 0.39 | 0.27 | 0.24 | 0.21 | 0.25 | 0.27 |
|  | Ray_f | 10.0 | 7.82 | 11.1 | 10.7 | 12.0 | 4.82 | 4.21 | 4.63 | 3.96 | 4.44 | 5.89 | 4.89 | 3.78 | 4.30 | 4.96 | 5.38 |
|  | N | 0.11 | 0.10 | 0.13 | 0.16 | 0.10 | 0.08 | 0.06 | 0.09 | 0.10 | 0.11 | 0.14 | 0.06 | 0.06 | 0.06 | 0.05 | 0.06 |
|  | P | 0.01 | 0.01 | 0.01 | 0.01 | 0.01 | 0.01 | 0.01 | 0.00 | 0.01 | 0.00 | 0.01 | 0.00 | 0.01 | 0.00 | 0.00 | 0.00 |
|  | pH | 5.37 | 5.28 | 4.22 | 4.04 | 5.48 | 7.18 | 4.86 | 5.05 | 4.45 | 5.49 | 5.12 | 5.16 | 4.95 | 4.31 | 4.43 | 5.06 |
|  | Phenol | 0.74 | 0.42 | 2.86 | 5.03 | 0.16 | 0.29 | 0.58 | 0.70 | 1.78 | 1.58 | 2.72 | 0.88 | 0.89 | 1.73 | 1.46 | 0.87 |
|  | Tannin | 0.43 | 0.29 | 2.45 | 4.44 | 0.06 | 0.17 | 0.39 | 0.40 | 1.01 | 1.22 | 2.05 | 0.56 | 0.60 | 1.50 | 1.29 | 0.64 |
|  | Wd | 0.60 | 0.52 | 0.64 | 0.59 | 0.66 | 0.45 | 0.39 | 0.42 | 0.35 | 0.37 | 0.61 | 0.39 | 0.38 | 0.52 | 0.49 | 0.43 |
|  | C | 45.1 | 45.5 | 46.2 | 47.6 | 46.3 | 46.3 | 45.9 | 49.8 | 49.6 | 50.6 | 50.1 | 47.4 | 46.7 | 47.8 | 47.6 | 47.9 |
|  | C/N | 423 | 467 | 365 | 315 | 462 | 581 | 871 | 601 | 492 | 483 | 369 | 777 | 789 | 885 | 975 | 858 |
|  | Lignin | 16.2 | 15.5 | 17.0 | 17.8 | 17.6 | 17.0 | 15.7 | 32.3 | 31.9 | 33.7 | 31.2 | 27.6 | 26.6 | 28.3 | 26.8 | 27.3 |
|  | Lign/cellu | 0.45 | 0.46 | 0.49 | 0.60 | 0.53 | 0.50 | 0.48 | 1.08 | 1.04 | 1.09 | 1.13 | 0.92 | 0.85 | 0.93 | 0.78 | 0.84 |
| Bark | N | 0.65 | 0.55 | 0.71 | 0.69 | 0.65 | 0.68 | 0.67 | 0.72 | 0.50 | 0.51 | 1.31 | 0.51 | 0.64 | 0.46 | 0.45 | 0.40 |
|  | P | 0.05 | 0.04 | 0.04 | 0.02 | 0.04 | 0.09 | 0.06 | 0.03 | 0.03 | 0.02 | 0.09 | 0.05 | 0.06 | 0.03 | 0.04 | 0.04 |
|  | pH | 5.22 | 5.16 | 4.64 | 4.27 | 5.31 | 5.93 | 4.97 | 4.36 | 4.85 | 4.08 | 5.22 | 4.95 | 4.79 | 4.42 | 4.45 | 4.56 |
|  | Phenol | 5.82 | 4.06 | 5.07 | 5.04 | 1.75 | 3.72 | 5.58 | 5.23 | 4.00 | 3.87 | 6.84 | 5.56 | 2.84 | 10.7 | 8.04 | 2.42 |
|  | Tannin | 3.49 | 2.09 | 3.41 | 3.67 | 1.12 | 2.91 | 3.84 | 4.15 | 2.84 | 3.26 | 5.50 | 3.85 | 1.77 | 6.75 | 4.89 | 1.71 |
|  | C | 46.2 | 52.4 | 46.2 | 49.0 | 46.9 | 47.8 | 48.3 | 48.9 | 47.4 | 49.2 | 45.5 | 49.3 | 49.9 | 52.9 | 52.8 | 49.2 |
|  | C/N | 72.2 | 97.2 | 65.3 | 72.2 | 72.8 | 71.5 | 74.7 | 69.0 | 95.9 | 99.1 | 35.9 | 97.7 | 79.0 | 118 | 122 | 124 |
|  | Lignin | 10.6 | 30.0 | 26.4 | 27.2 | 24.0 | 25.5 | 21.5 | 32.8 | 27.5 | 35.0 | 18.2 | 32.8 | 34.9 | 36.5 | 39.4 | 31.2 |
|  | Lign/cellu | 0.58 | 1.53 | 1.28 | 1.48 | 1.13 | 1.08 | 1.44 | 1.26 | 1.08 | 1.47 | 1.17 | 1.44 | 1.52 | 1.76 | 2.04 | 0.97 |
|  | B_resistance | 84.9 | 111 | 72.3 | 75.5 | 59.1 | 73.2 | 95.0 | 35.0 | 46.8 | 37.5 | 33.9 | 55.9 | 51.7 | 48.9 | 42.6 | 73.1 |

**Supplementary Table 5.** Trait variation of wood and bark across 14 temperate tree species.

**Supplementary Figure 1.** The percentage of trait variation explained by major taxa (angiosperms vs gymnosperms), species within major taxa, compartments (innerwood, outerwood and bark) and stem length between sampled disk and tree top (yellow).


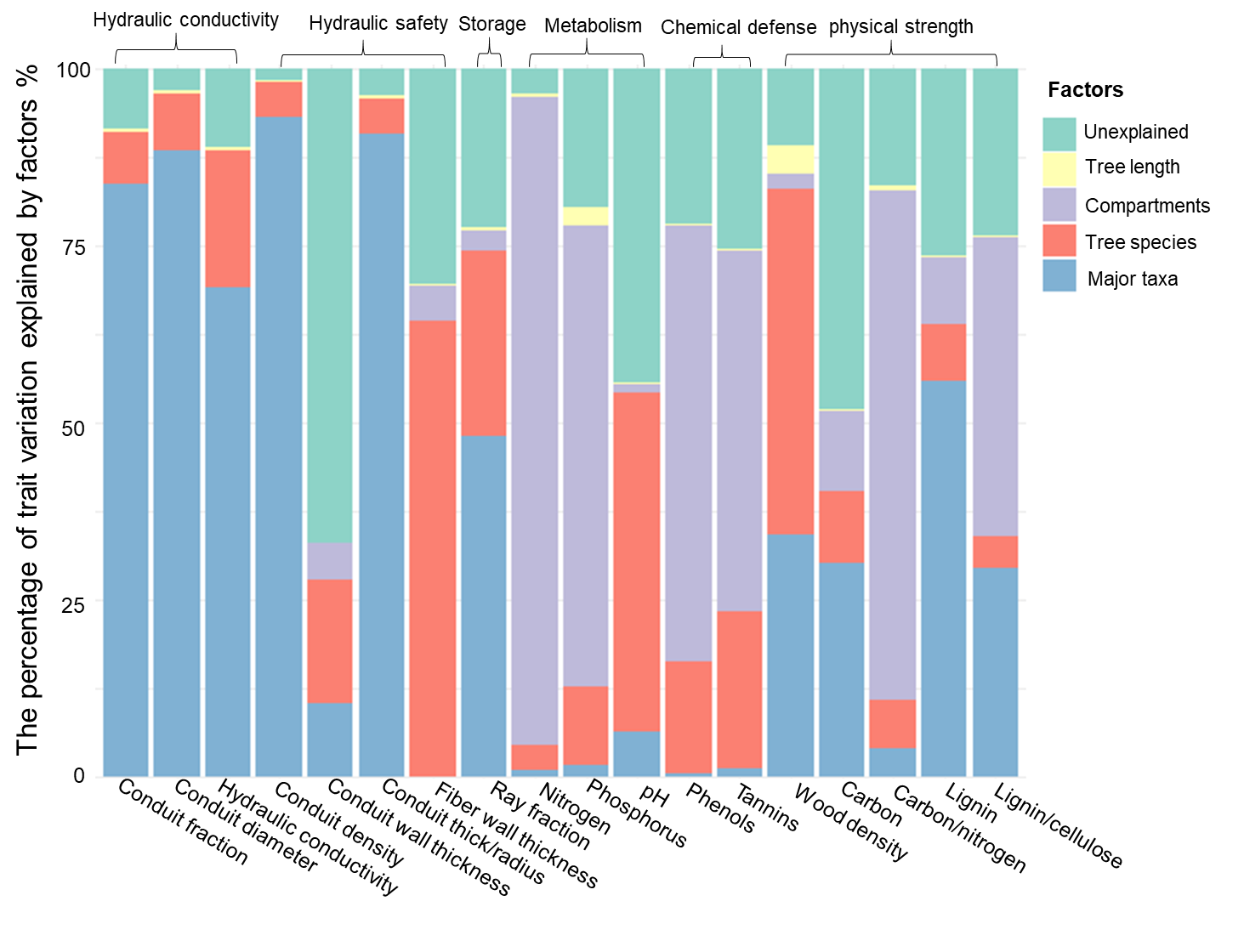


**Supplementary** **Figure 2.** Covariance matrix showing the associations of all stem traits in different tissues of **all species**. Only significant correlations (p<0.05) were shown here. Red dots indicate negative correlations, blue dots refer to positive correlations, and the darker of the color the stronger the correlation. See Table S1 for trait abbreviations.


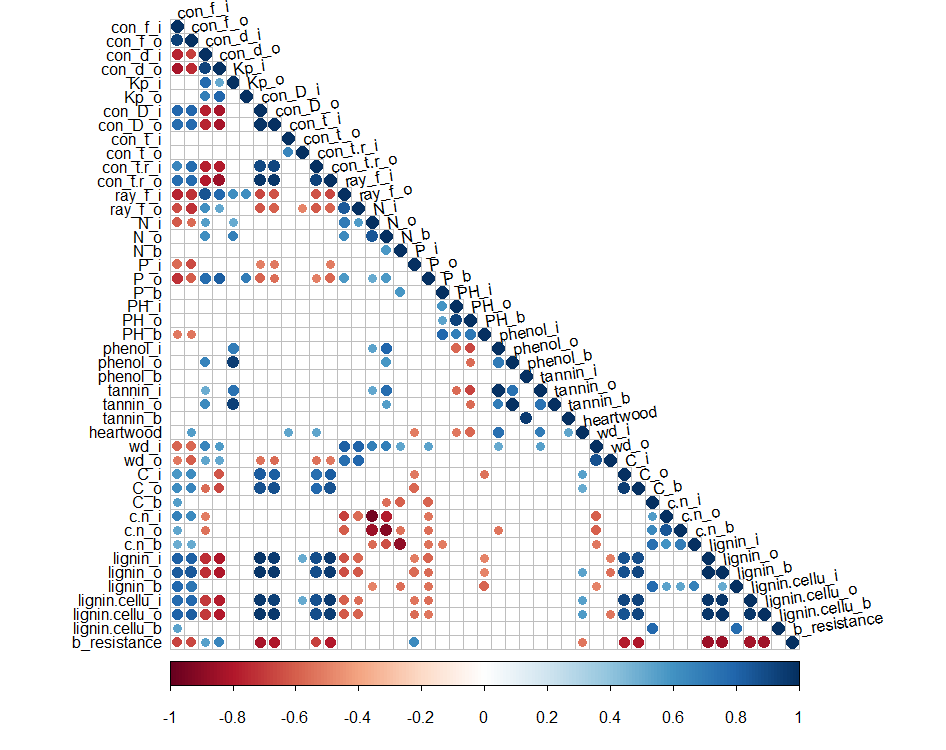


**Supplementary Figure 3.** Covariance matrix showing the associations of all stem traits in different tissues of **angiosperms**. Only significant correlations (p<0.05) were shown here. Red dots indicate negative correlations and blue dots refer to positive correlations. The darker of the color, the stronger the correlation. See Table S1 for trait abbreviations.


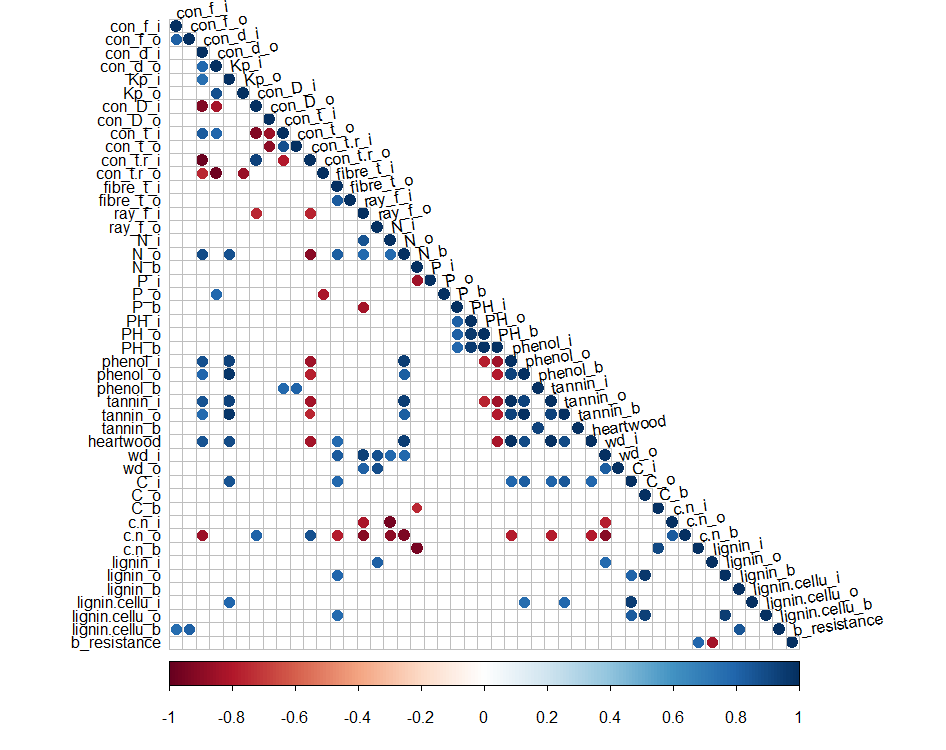


**Supplementary Figure 4.** Covariance matrix showing the associations of all stem traits in different tissues of **gymnosperms**. Only significant correlations (p<0.05) were shown here. Red dots indicate negative correlations and blue dots refer to positive correlations. The darker of the color, the stronger the correlation. See Table S1 for trait abbreviations.


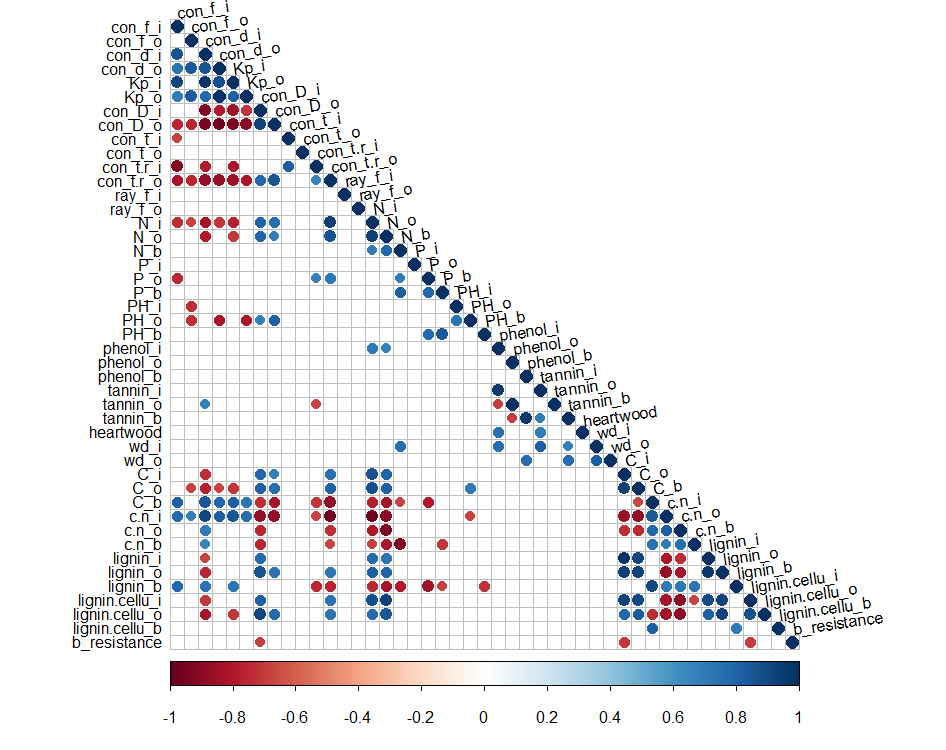


# **References**

Chave J, Coomes D, Jansen S, Lewis SL, Swenson NG, Zanne AE (2009) Towards a worldwide wood economics spectrum. Ecol Lett 12: 351-366. <https://doi.org/10.1111/j.1461-0248.2009.01285.x>

Sattler S, Funnell-Harris D (2013**)** Modifying lignin to improve bioenergy feedstocks: strengthening the barrier against pathogens?. Front. Plant Sci. **4**: 70. <https://doi.org/10.3389/fpls.2013.00070>

Wainhouse D, Cross DJ, Howell RS (1990) The role of lignin as a defense against the spruce bark beetle Dendroctonus micans: effect on larvae and adults. Oecologia **85**(2): 257-265. <https://doi.org/10.1007/BF00319411>

Zanne AE, Westoby M, Falster DS, Ackerly DD, Loarie SR, Arnold SE, Coomes DA (2010) Angiosperm wood structure: global patterns in vessel anatomy and their relation to wood density and potential conductivity. Am J Bot 97: 207-215. <https://doi.org/10.3732/ajb.0900178>

Valette N, Perrot T, Sormani R, Gelhaye E, Morel-Rouhier M (2017) Antifungal activities of wood extractives. Fungal Biol Rev 31(3): 113-123. <https://doi.org/10.1016/j.fbr.2017.01.002>

Mounguengui S, Tchinda JBS, Ndikontar MK, Dumarçay S, Attéké C, Perrin D, Gelhaye E,

Gérardin P (2016) Total phenolic and lignin contents, phytochemical screening, antioxidant and fungal inhibition properties of the heartwood extractives of ten Congo Basin tree species. Ann For Sci 73: 287-296 . <https://doi.org/10.1007/s13595-015-0514-5>

Rektorschek M, Weeks D, Sachs G, Melchers K (1998) Influence of pH on metabolism and urease activity of Helicobacter pylori. Gastroenterology 115 (3): 628-641. <https://doi.org/10.1016/S0016-5085(98)70142-8>

Zhong C, Cao X, Hu J, Zhu L, Zhang J, Huang J, Jin Q (2017) Nitrogen metabolism in adaptation of photosynthesis to water stress in rice grown under different nitrogen levels. Front Plant Sci 8: 1079. <https://doi.org/10.3389/fpls.2017.01079>

Janssen TA, Hölttä T, Fleischer K, Naudts K, Dolman H (2020) Wood allocation trade‐offs between fiber wall, fiber lumen, and axial parenchyma drive drought resistance in neotropical trees. Plant Cell Environ 43(4): 965-980. <https://doi.org/10.1111/pce.13687>

Jacobsen AL, Ewers FW, Pratt RB, Paddock WA, Davis SD (2005) Do xylem fibers affect vessel cavitation resistance ?. Plant Physiol 139 (1): 546-556.  <https://doi.org/10.1104/pp.104.058404>

Dimond AE (1966) Pressure and flow relations in vascular bundles of the tomato plant. Plant Physiol 41: 119-131. <https://doi.org/10.1104/pp.41.1.119>

Sterck FJ, Zweifel R, Sass-Klaassen U, Chowdhury Q (2008) Persisting soil drought reduces leaf specific conductivity in Scots pine (Pinus sylvestris) and pubescent oak (Quercus pubescens). Tree Physiol 28: 529-536. <https://doi.org/10.1093/treephys/28.4.529>

Martínez-Cabrera HI, Estrada-Ruiz E (2014) Wood anatomy reveals high theoretical hydraulic conductivity and low resistance to vessel implosion in a Cretaceous fossil forest from northern Mexico. PloS one 9(10): e108866. <https://doi.org/10.1371/journal.pone.0108866>

Rasmann S, Bauerle TL, Poveda K, Vannette R (2011) Predicting root defence against herbivores during succession. Funct Ecol 25(2): 368-379. <https://doi.org/10.1111/j.1365-2435.2010.01811.x>
